# Parent-young communication on sexual and reproductive health issues and its associated factors: experience of students in Agaro Town, Ethiopia

**DOI:** 10.1186/s12978-022-01553-0

**Published:** 2023-01-06

**Authors:** Daba Abdissa, Workitu Sileshi

**Affiliations:** 1 Department of Biomedical Sciences, College of Medical Sciences, Institute of Health Science, Jimma University, Jimma, Ethiopia; 2 Department of Midwifery, College of Health Sciences, Institute of Health Science, Jimma University, Jimma, Ethiopia

**Keywords:** Communication, Sexual and reproductive health, Young population, Parent, Ethiopia

## Abstract

**Background:**

Sexual and reproductive health (SRH) is at the base of young people's living and wellbeing. A significant number of young peoples are affected by avoidable SRH problems due to a lack of appropriate knowledge regarding SRH. Parent-young communication on SRH is critical in informing them about risk and protective behaviors which in turn decrease the likelihood of involvement in risky sexual behaviors. Therefore, the purpose of this study was to evaluate the parent-young communication on SRH issues and associated factors among secondary and preparatory school students at Agaro town, Southwestern Ethiopia.

**Methods:**

School based cross-sectional study was conducted from April 13 to April 20, 2019 using stratified random sampling technique. A total of 315 students were included to the study. Data were collected using pretested interviewer-administered structured questionnaire entered into Epi data version 3.1; and analyzed using SPSS version 20. A variable having a p-value of < 0.25 in the bivariable logistic regression model was subjected to multivariable logistic regression analysis to avoid the confounding variable’s effect. Adjusted odds ratios were calculated at the 95% confidence interval and considered significant with a p-value of < 0.05.

**Results:**

The mean age of the respondents was 20.2 ± 2.6 years. The study finding showed that 61.3% of the participants were discussed on SRH issues with their parents. According to multivariate analysis; educational status of mother [primary education (AOR = 3.67; 95%CI = 1.93, 6.97),secondary education(AOR:2.86; 95%CI = 1.20, 6.80)],educational status of father[primary education (AOR = 5.8;95%CI = 2.8, 12.3,secondary education (AOR = 3.21; 95%CI = 1.55, 6.59)],having family size of < 5 (AOR = 6.4; 95%CI = 3.36, 12.37) and having boy/girlfriend(AOR = 1.99; 95%CI = 1.0, 3.8) were significantly associated with parent-young people communication.

**Conclusion:**

About two third of the participants communicate with their parents about SRH issues and parents’ educational status, family size of < 5 and having boy/girlfriend were significantly associated with it. The main reasons for not communicated was cultural taboos, shame and parents lack of knowledge. Therefore, it is necessary to educate and equip students and parents to address the identified problems.

**Supplementary Information:**

The online version contains supplementary material available at 10.1186/s12978-022-01553-0.

## Plain English summary

Sexual and reproductive health (SRH) is at the base of young people's living and wellbeing. A significant number of young peoples are affected by avoidable SRH problems. Parent-young communication on SRH is critical in informing them about risk and protective behaviors which in turn decrease the likelihood of involvement in risky sexual behaviors. This study was intended to assess this issue on students learning at Agaro town, Ethiopia during April 13 to April 20, 2019 using pretested interviewer-administered structured questionnaire with sample size of 315.

The study finding showed that 61.3% of the participants were discussed on SRH issues with their parents. According to multivariable logistic regression analysis educational status of parents, having family size of < 5 and boy/girlfriend were significantly associated with parent adolescent communication. Cultural taboos, shame and parents lack of knowledge were the major barriers for communication. Thus, it is necessary to educate students and parents to address the identified problems thereby prevent young population from risky sexual behaviors.

**Supplementary Information:**

The online version contains supplementary material available at 10.1186/s12978-022-01553-0.

## Introduction

Globally, there are 1.8 billion young people (age 10–24 years) and 90% of them live in developing countries [1, 2]. Young age is a critical stage of rapid biological and psychosocial changes that affect all aspects of life, and it is also an important period to lay the foundations of good health in adulthood [3]. Unsafe abortion, early pregnancy, high rates of sexually transmitted infections (STIs), difficulty accessing contraception, cultural factors and socio-economic are some of the challenges faced by young people [4].

Each year around the globe sixteen million late adolescent girls give birth, in which 95 percent of them occur in developing countries [5]. Young people usually engage in risky sexual behaviors and highly affected by the burden of unwanted pregnancy, HIV/AIDS, STIs and other reproductive ill health due to lack of awareness about risky sexual behaviors [6–8]. Globally greater than 60% of young people were living with HIV, and they also account for 58% of newly acquired HIV infections [9]. Unplanned and unwanted pregnancy for unmarried young women results in dropout of school, rejection from family and community. The main cause for the above situation is the gap in promoting the sexual and reproductive health (SRH) agenda and young population health is usually ignored area in health priority [10].

One of the priorities of the Sustainable Development Goals is improving the SRH of the young population [11]. To this end, Ethiopia has also developed a strategy specifically targeting young people [12]. However, There are only few national programs specifically targeted to addressing the needs of this group and the problem of SRH among young people remains a challenge [13]. Moreover, lack of integration and shortage of young-friendly services are common problems in most developing countries, including our country [4].

Parent-young population communication on SRH issues is vital in reducing risky sexual behaviors and negative its consequences [14]. It is fundamental process through which parents transmit sexual values, information, beliefs, and expectations to their children with the purpose of influencing sexual behaviours, attitudes and decision-making of their children [15]. Parent-young population communication is one potential source of SRH information for young people [16]. Evidence revealed that 29% of premarital sex is due to inappropriate parenting [17]. The Ethiopian government has established a strategic goal to encourage parental participation, but little is known about parental participation [18] and the role and current status of parent-young population discussions on SRH issues have not been well addressed yet [19].

Reasons for not discussing with parents include sociocultural norms, lack of knowledge and parental fear [20]*.* As a result, most of the young people attempts regarding sexual matters is generally misguided by their peer group of the same sex and many teenagers do not have access to reliable information regarding their SRH needs. A significant number of young peoples are affected by avoidable SRH problems due to a lack of appropriate knowledge regarding SRH. Parent-young communication on SRH is critical in informing them about risk and protective behaviors [6, 21]. According to previous studies, the factors associated with the parent-young people communication about SRH were parental education, adolescent’s age and living arrangements, type of parents, and parents’ SRH knowledge and attitude [20, 22, 23] (Additional file 1).

Generally, there is inadequate evidence about the proportion of parent-young people communication in Ethiopia [24], point prevalence varies among the studies and there is no established evidence at the study area. Moreover, there are inconsistent findings with regard to proportion and factors associated with parent and young people communication on SRH issues. For instance, according to study finding gender had a positive relationship with communication on SRH [22, 25] while other studies showed no association [6].

Hence, this study was aimed at assessing the proportion and associated factors of parent young communication on SRH among secondary and preparatory school students for risk minimization and better health at the study area. This study will help to provide information regarding the proportion of parent young people communication on SRH and its determinants which helps as an input to design appropriate intervention programs. Furthermore, this study was attempted to generate evidence based information for concerned government bodies and policy makers to consider the situation and to design an appropriate intervention strategy.

## Methods and materials

### Study area

The study was conducted at Agaro town, Jimma Zone, Southwest Ethiopia. It is located 45 km from Jimma town and 391 km from Addis Ababa. There are educational services from kindergarten to university in the town and it has two health centers and one primary hospital. There are 2 secondary and preparatory schools and 4 secondary schools in the town. According to the data obtained from school directors, the school had 2818 total regular secondary and preparatory students during the study period.

### Study period

The study was conducted from April 13 to April 20, 2019.

### Study design

A descriptive facility based cross-sectional study was conducted among secondary and preparatory school students at Agaro town.

### Study population

The source population comprised of all Agaro secondary and preparatory school students whose age was 10–24 years during the academic year of 2019, while the study population comprised all Agaro secondary and preparatory school students who fulfilled the inclusion criteria.

### Eligibility criteria

Regular students were included in the study, whereas students absent from class on data collection days live alone, orphans or live with guardian who are not their biological parents, deaf, dumb students, sick and married students were excluded.

### Sample size determination

The sample size was determined using single population proportion formula considering the following assumptions: P = 28.9% [26], significance level 5% and margin of error 5%.

$$n=\frac{(z \alpha \left/ 2 \right. )^2 p (1-p)}{(d)^2}=\frac{ (1.96)2 0.289(1-0.289)}{(0.05)^2}=317,$$

where n = required sample size, Z = Percentiles of the standard normal distribution corresponding to 95% confidence level assumption, $z \alpha \left/ 2 \right.$= Coefficient at level of significance = 1.96, p = 28.9% proportion of parent young communication (according to study done in Mizan), d = Margin of error = 0.05.

Accordingly it gives initial sample size of 317. Since the source population is less than 10,000, (2818), we employed population correction formula for a finite population.

$${n}_{f}=\frac{n}{1+\frac{n}{N}}\rightarrow\frac{317}{1+\frac{317}{2818}}\rightarrow{ n}_{f}=286,$$

where*: *N = sample size. N = total population (2818). nf = final sample size.

By taking into consideration 10% non-response rate, the final sample size was 286 + 28.6 = 315.

### Sampling procedure

**Fig. 1:**
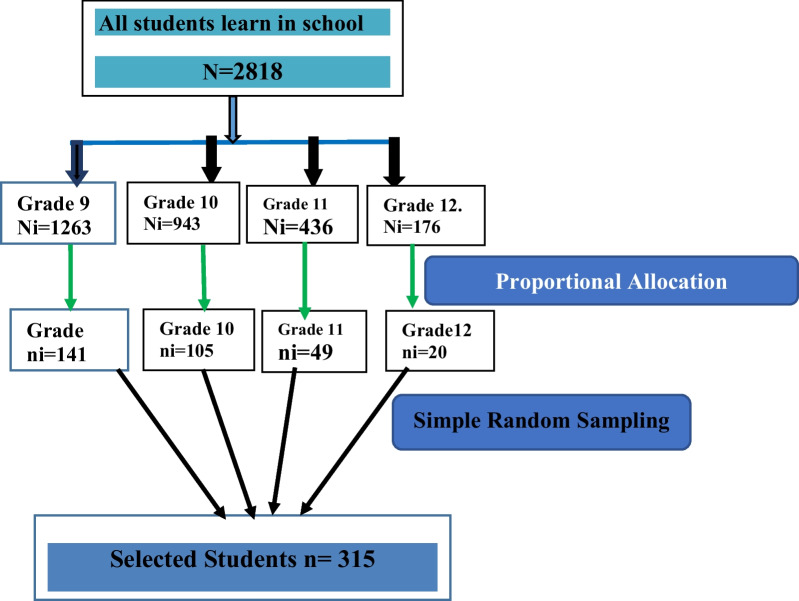
Schematic diagram representation of sampling procedure for selecting participants at Agaro secondary and preparatory school, Southwest Ethiopia 2021

Stratified random sampling technique was employed to select study participants. First the sampling frame was prepared by having lists of students from grade 9 to 12 and then the sample population was proportionally assigned to each grade. Proportional allocation was done by allocating sampling proportional to the total population of each unit, using the formula:

$$ni = \frac{n}{N} \times {\text{Ni,}}$$

where ni = sample size of students from each grade, n = total sample size of students, N = total population of students, and Ni = total population of students at each grade. Finally samples were selected from each class by simple random sampling technique using students’ roster (Fig. 1).

### Operational definition

Parents: in this study mean biological parents, step parents or foster parents but does not include elder siblings [27].

Parent-young communication on SRH: Students open discussion on at least two SRH issues (STIs/ HIV/AIDS, condom, sexual intercourse, premarital sex, puberty, menstrual cycle, unwanted pregnancy and contraception) with their parents in the last 12 months initiated by the young population or both [28].

Secondary school students: students who were in grade 9 and 10.

Preparatory school students: students who were in grade 11 and 12.

Regular students: students who were assigned on working day (from Monday to Friday) on regular working hour.

Young people: In this study context are peoples who are between 10–24 year olds.

Ever got SRH information = in this study context participants who had SRH information on at least two SRH issues during their life time.

Residence: In our study context place where students live for at least 6 months.

Ethnicity: nation of the study participant they belongs.

### Data collection instrument

Data were collected using pretested, validated, self-administered structured questionnaire which was developed through reviewing different related scientific literatures [6, 25, 26, 29] and it was pre-tested before collecting data to ensure data quality. The data were collected by six diploma graduate nurses under the supervision of two supervisors and principal investigator. Data collectors were supervised by two diploma midwives.

### Data quality control

Data quality was ensured through standardized data collection materials and the English version questionnaire was translated to local language (Oromic) version for appropriateness and easiness by language experts in both cases. The Oromic version was again translated back to English language to verify the content validity of the original version.

Two days of training was given for data collectors about the aim of the study, how to approach the study subjects, sampling procedure and the content of the questionnaire. The questionnaires were pre-tested on 5% of participants at the Jiren secondary and preparatory school one week ahead of actual data collection and further modified based on the results. Continuous follow-up and supervision were made by the two supervisors and principal investigator and collected data were reviewed and checked daily for clarity, completeness and consistency.

### Data entry, processing and analysis

The collected data was compiled, reviewed, coded and entered in to Epidata version 3.1 and exported to SPSS version 20 for analysis. Data was checked and cleaned for its completeness and errors in coding and entering before analysis. Descriptive statistical analysis was used to compute frequency and a percentage of independent and dependent variables. A logistic regression model was computed to see the association of independent variables and dependent variables. Variables with *p* ≤ 0.25 on bivariable logistic regression were considered as candidates for multivariable regression and *P*-values of < 0.05 were considered to be statistically significant in the multivariable analysis. Crude and adjusted odds ratios with their 95% confidence intervals were calculated. The Hosmer and Lemeshow goodness-of-fit test were checked and gave a *p* value of 0.704, indicating evidence of fitness of the model. The variance inflation factor was used to verify the multico-llinearity between the independent variables, and no findings were found.

## Results

### Socio demographic characteristics of the study population

A total of 315 students were included to the study. The mean age of the respondents was 20.2 ± 2.6 years. Regarding religion most of the respondents 263(83.5%) were muslim. One hundred forty one (44.7%) was grade nine students followed by grade ten students who account 105 (33.3%). Most of young students lives with both parents 279(88.6%). Almost half (53.3%) of the participants had ever got SRH information (Table 1).

**Table 1:** Socio demographic characteristics of Agaro secondary and preparatory school students and their parents, Agaro town, Southwest Ethiopia, 2019

Variables	Number	Percent
Sex		
Male	150	47.6
Female	165	52.4
Age (years)		
15–19	142	45.1
20–24	173	54.9
Grades		
9	141	44.7
10	105	33.3
11	49	15.6
12	20	6.4
Ethnicity		
Oromo	263	83.5
Amhara	29	9.2
Others^a^	23	7.3
Residence		
Urban	112	35.6
Rural	203	64.4
Have boy/girlfriend		
Yes	83	26.3
No	232	73.7
Had ever got SRH information		
Yes	168	53.3
No	147	46.7
Religion		
Muslim	263	83.5
Orthodox	31	9.8
Others^b^	21	6.7
Living arrangement of adolescents		
With both parents	279	88.6
With one parent	17	5.4
With relative	11	3.5
Marital status of parents		
Alone	8	2.5
Together	295	93.7
Widowed	14	4.4
Divorced	6	1.9
Mother’s educational status		
Illiterate	91	28.9
Primary school	150	47.6
Secondary school and above	74	23.5
Father's educational status		
Illiterate	73	23.2
primary school	134	42.5
Secondary school and above	108	34.3
Occupation of family		
House wife	71	22.5
Farmer	108	34.3
Employed	58	18.4
Private	64	20.3
Others	14	4.4
Family size		
< 5	175	55.6
≥ 5	140	44.4
Estimated family income per month (in birr)		
< 1000	34	18.9
1000–2000	81	45
≥ 2000	65	36.1
I don’t know	135	42.9

^a^Wolayta, Dawuro, Tigre

^b^Retired, unemployed

### Communication on sexual and reproductive health issues

Although 92% of the respondents reported that it was important to discuss SRH issues with parents, only 61.3% had communicated with their parents on at least two SRH topics. The major topics discussed among participants were premarital sex (40%) followed puberty (37.8%) (Table 2).

**Table 2:** Different SRH issues discussed by secondary and preparatory school students in Agaro town, Southwest Ethiopia, 2019

SRH issues discussed	Category	Total (n, %)
Important to discuss on SRH issues	Agree	290 (92)
	Neutral	2.5
	Disagree	5.5
Discussion on at least two SRH issues	Yes	193 (61.3)
	No	122 (38.7)
Contraceptive	Yes	62 (19.7)
	No	253 (80.3)
Menstrual cycle	Yes	67 (21.3)
	No	248 (78.7)
STIs/HIV/AIDS	Yes	112 (35.6)
	No	203 (64.4)
Unwanted pregnancy	Yes	75 (23.8)
	No	240 (76.2)
Premarital sex	Yes	126 (40)
	No	189 (60)
Abortion	Yes	29 (9.3)
	No	286 (90.7)
Puberty	Yes	119 (37.8)
	No	196 (62.2)
Sexual intercourse	Yes	45 (14.3)
	No	270 (85.7)
Condom	Yes	36 (11.4)
	No	279 (88.6)

Only hundred twelve (35.6%) of the students had discussed about STIs/HIV/AIDS because of parents’ lack of knowledge (49.5%) and shame (35.9%). Similarly only 23.8% of the students discussed with their parents about unwanted pregnancy due to shame (46%) and parents lack of (24.8%) (Table 3).

**Table 3:** Reasons for young students for not discussing on SRH issues with their parents in Agaro town, Southwest Ethiopia, 2019

Topic of discussion	Not discussed (n, %)	Reasons for not discussing					
		Shame^a^	Culturally unacceptable^a^	Parents lack knowledge^a^	Parents too busy^a^	Difficult and embar-rassing^a^	Others^b^
Contraceptive	253 (80.3)	129 (40.9)	84 (26.7)	95 (30.2)	46 (14.6)	54 (17.1)	8 (2.5)
STIs/HIV/AIDS	203 (64.4)	113 (35.9)	76 (24.1)	156 (49.5)	37 (11.7)	31 (9.8)	11 (3.4)
Unwanted pregnancy	240 (76.2)	145 (46)	32 (10.2)	78 (24.8)	27 (8.5)	64 (20.3)	7 (2.2)
Premarital sex	189 (60)	178 (56.5)	57 (18.1)	41 (13)	19 (6)	94 (29.8)	13 (4.1)
Sexual intercourse	270 (85.7)	190 (60.3)	31 (9.8)	51 (16.2)	12 (3.8)	75 (23.8)	15 (4.8)
Condom	279 (88.6)	201 (63.8)	74 (23.5)	25 (7.9)	13 (4.1)	87 (27.6)	11 (3.5)
Puberty	196 (62.2)	82 (26)	11 (3.5)	32 (10.2)	43 (13.7)	18 (5.7)	7 (2.2)

^a^Multiple responses were possible

^b^Belief that it initiate sex, religious belief, don’t now

### Factors associated with parent-young communications on SRH issues

Bivariable logistic regression analysis was performed to assess association between each independent variable and outcome variable. Results of bivariable analysis showed that sex of student, mother education status, father education status, age, having ever got SRH information, having boy/girlfriend, residence, occupation of family and family size show association with parent-young communication. All of them were entered to multivariable analysis to control effect of confounding. The result of multivariable logistic regression model revealed that educational status of biological parents, family size of < 5 and having boy/girlfriend were significantly associated with parent adolescent communication.

The odds of parent-young communication were 6.4 times [AOR = 6.4; 95% CI: 3.36, 12.37] higher among students of family size of < 5 than their counter parts. The odds of parent-young communication were 1.99 times [AOR = 1.99; 95% CI: 1.3.0, 8.0] higher among students who had boy/girlfriend than those who had no boy/girlfriend.

Young students, whose mothers had primary education, were 3.67 times more likely to communicate on SRH issues with their parents than those students whose mothers were illiterate (AOR = 3.67; 95%CI:1.93–6.97). Similarly, young students whose mothers had secondary education were 2.86 times more likely to communicate on SRH issues with their parents than those students whose mothers were illiterate (AOR = 2.86; 95%CI: 1.2, 6.8).This study also revealed fathers’ education [primary education (AOR = 5.8 95%CI: 2.8, 12.3),secondary education (AOR = 3.21 95%CI:1.55, 6.59)]were significantly associated with parent-young communication after controlling for confounders (Table 4).

**Table 4:** Bivariable and multivariable logistic regression analysis for factors associated with parent-young communication among participants in Agaro town, Ethiopia 2019

Variables	Category	Parent-young communication		Bivariable analysis		Multivariable analysis	
		Yes	No	P-value	COR (95%CI)	P-value	AOR (95%CI)
Age (years)	15–19	82	60	1	1	1	
	20–24	111	62	0.245	1.31 [0.83, 2.1]	0.911	1 [0.58, 1.81]
Mother educational level	Illiterate	36	55	1	1	1	1
	Primary	109	41	0	4 [2.3, 7]	≤ 0.001	3.67 [1.93, 6.97]
	Secondary	48	26	0.001	2.8 [1.49, 5.33]	0.017*	2.86 [1.2, 6.8]
Father educational status	Illiterate	28	45	1	1	1	1
	Primary	96	38	≤ 0.001	4 [2.2, 7.4]	≤0.001*	5.8 [2.8, 12.3]
	Secondary	69	39	0.001	2.8 [1.54, 5.25]	0.002*	3.2 [1.55, 6.59]
Occupation of family	Housewife	40	31	1	1	1	1
	Farmer	61	47	0.985	1 [0.55, 1.84]	0.613	1.2 [0.58, 2.45]
	Employed	37	21	0.391	1.36 [0.67, 2.78]	0.676	1.19 [0.52, 2.73]
	Private worker	45	19	0.095	1.83 [0.9, 3.74]	0.108	2 [0.85, 4.77]
	Other^a^	10	4	0.300	1.93 [0.55, 6.76]	0.955	1 [0.25, 4.3]
Sex	Male	101	49	0.036	1.636 [1, 2.58]	0.100	2.45 [0.84, 6.89]
	Female	92	73	1	1	1	1
Residence	Rural	115	88	1	1	1	1
	Urban	78	34	0.024	1.75 [1, 2.86]	0.774	0.86 [0.32, 2.34]
Family size	< 5	129	46	≤ 0.001	3.3 [2, 5.34]	≤ 0.001*	6.4 [3.36, 12.37]
	≥ 5	64	76	1	1	1	1
Has boy/girlfriend	Yes	59	24	0.034	1.79 [1, 3]	0.041*	1.99 [1, 3.8]
	No	134	98	1	1	1	1
Had ever got SRH information	Yes	110	58	0.102	0.68 [0.43, 1]	0.260	0.73 [0.43, 1.26]
	No	83	64	1	1	1	1

*Value statistically significant

^a^Retired, unemployed

*AOR* adjusted odds ratio, *COR *crude odds ratio; 1-reference

## Discussion

This study has attempted to assess the proportion of parent-young communication on SRH issues and its associated factors in Agaro secondary and preparatory school students, Southwest Ethiopia. The magnitude of parental communication on SRH issues was reported to be only 61.3% (193) [95% CI: 55.6, 66.7]. This finding was in line with two independent studies done in Ethiopia, 59.1% in Yergalem, South Ethiopia [29] and 57.6% in Mekelle, Northern Ethiopia [30]. However, this result was higher than three independent studies done in Ethiopia: 48.5% in Dabat, Northwest Ethiopia [31], 47% in Robe, Southeast Ethiopia [32] and 37% in Dire Dewa, Eastern Ethiopia [6]. Those differences might be due to the difference in socio-demographic, cultural difference and accessing sexual reproductive health information. For example, the possible explanation of higher prevalence in our study could be due to home to home, school and mass health education and information by Jimma University health science students during community based team training program every year.

In contrast, the finding of the current study was lower than that of a study conducted in Hayik and Wolaita Sodo, Ethiopia were 83% [25] and 85%, respectively [33]. The possible reason for the difference in our finding and the one from Wolaita Sodo could be due to the difference in the study population, i.e., the study from Wolaita Sodo included only female university students with large sample size who have better information. Similarly in the study conducted in Hayk, more than three-quarters of the participants lived in urban areas with adequate access to reproductive health services, and all participants were from preparatory schools. Moreover, the study from Ghana reported 82.3% [34]. The difference might be related to difference in sample size used, sampling technique and culture related to openness related to SRH issues.

The major topics discussed among participants were premarital sex, puberty and STIs/HIV/AIDS, while topics like contraception was discussed less among participants. The possible justification might be as majority of the participants were Muslim followers and their religion did not encourage them to use contraception including condoms which might affect their discussion. Thus, we recommend future study supported with qualitative findings to explore on this issue.

Regarding challenges for not discussing SRH issues shame, cultural taboos, embarrassments and parents' lack of knowledge were mentioned by the majority of participants. For instance, almost three quarters of participants were not discussed regarding unwanted pregnancy due to shame and parents’ lack of knowledge 46% and 24.8%, respectively. This was consistent with previous studies [25, 27]. This might be due to the fact that sexual conversations are deemed a taboo subject in many African communities, including our country. Moreover, parent-young communication on SRH issues in Ethiopia is believed to be socially disgraceful and parents are not open and uncomfortable to discuss these issues with their young children [35].

In agreement with earlier studies, in this study educational status of father and mother showed statistical significance with parent adolescent communication about SRH issues [25, 36, 37]. This could be due to educated parents have better access to health service information, improved perceptions of SRH issue and better skill of communication.

In the same manner, the study showed that those students whose family size was less than five were more likely to communicate which agreed with previous study [38]. This result demonstrated that those parents with small family size had better chance of discussing SRH issues with their children. Finally, the present study revealed that there were significant association between parent adolescent’s sexual and reproductive issues and having boy/girlfriend. The possible reason for this association might be students who enter to relationship might raise about SRH issues with each other and they may discuss with their parents for more information.

### Limitation of the study

1st due to cross-sectional nature of the study, it was difficult to imply cause–effect relationship. 2^nd^ this study was done to assess parent young-people communication were based on young population perceptions, which may not reflect what parents were actually doing. 3rd, since it was based on self-reporting, it might be affected by social desirability bias because of sensitive nature and cultural barrier for open discussion and finally additional qualitative study should be done to explore more on young and parents communication.

## Conclusion

This study showed about two third of young population had discussion on SRH issues with their parents on at least two SRH issues. Educational level of parents, having family size of < 5 and having boy/girlfriend were significantly associated with communication. The main reasons for not communicated was cultural taboos, shame and parents lack of knowledge. Thus, it is necessary to educate and equip students and parents to address the identified problems.

Students and their parents should receive health education on the significance of talking about SRH issues and related consequences of risky sexual behaviors. To promote and support school sex education, teachers should receive SRH training. Moreover, more research is required to identify barriers especially with regard to parents.

In order to build a supportive atmosphere for parent-young people communication regarding SRH, policy makers will need to design tailored action to create supportive environment for parent young people communication concerning SRH.

## Supplementary Information

**Additional file 1: Figure S1.** Conceptual framework showing the possiblefactors that could contribute to the Parent young communication [18, 20, 22, 23, 25, 35–37].

## Data Availability

The data used to support the findings of this study can be available from the corresponding author upon reasonable request.
