# Supplementary material for: Parent-young communication on sexual and reproductive health issues and its associated factors: experience of students in Agaro Town, Ethiopia
Source: Reprod Health. 2023 Jan 6;20:11. doi: 10.1186/s12978-022-01553-0 (PMC9824963; doi:10.1186/s12978-022-01553-0)
Supplement: Supplementary file 1 — Additional file 1: Figure S1. Conceptual framework showing the possiblefactors that could contribute to the Parent young communication [18, 20, 22, 23, 25, 35–37]. [file 12978_2022_1553_MOESM1_ESM.docx]

agesexgradeethnicityreligionResidence agesexgradeethnicityreligionResidence Marital status of ParentsMother’s educational statusFather's educationalStatusOccupation of familyFamily sizefamilyincomeMarital status of ParentsMother’s educational statusFather's educationalStatusOccupation of familyFamily sizefamilyincome


                                                                                                       


   Parent-young communication   Parent-young communication
	

	

Have boy/girlfriendHad ever got SRH informationliving arrangement parents SRH knowledge parents SRH attitudeHave boy/girlfriendHad ever got SRH informationliving arrangement parents SRH knowledge parents SRH attitude


Figure 1: Conceptual framework showing the possible factors that could contribute to the Parent young communication [18, 20, 22, 23, 25, 35-37]
   Utero vaginal prolapse (UVP)   Utero vaginal prolapse (UVP)
